# Dysregulation of autophagy in human follicular lymphoma is independent of overexpression of BCL-2

**DOI:** 10.18632/oncotarget.2605

**Published:** 2014-10-18

**Authors:** Aine McCarthy, Jacek Marzec, Andrew Clear, Robert D. Petty, Rita Coutinho, Janet Matthews, Andrew Wilson, Sameena Iqbal, Maria Calaminici, John G. Gribben, Li Jia

**Affiliations:** ^1^ Centre for Haemato-Oncology, Barts Cancer Institute, Queen Mary University of London, London, United Kingdom; ^2^ Centre for Molecular Oncology2, Barts Cancer Institute, Queen Mary University of London, London, United Kingdom

**Keywords:** Autophagy, BCL-2, follicular lymphoma, autophagy PCR array, tissue microarray

## Abstract

Overexpression of the anti-apoptotic protein BCL-2 is characteristic of human follicular lymphoma (FL) and some cases of diffuse large B cell lymphoma (DLBCL). We aimed to determine autophagy status in primary FL and DLBCL samples and the BCL-2^+^/BCL-2^−^ lymphoma cell lines using both autophagy PCR array and tissue microarray (TMA). A greater number of autophagy machinery genes were up-regulated in the BCL-2^+^ Su-DHL4 cell line compared with BCL-2^−^ Su-DHL8 cells, at both the basal level and in response to autophagic stress. The autophagy-related gene expression profiles were determined in purified and unpurified malignant human lymph node biopsies. Seven autophagy machinery genes were up-regulated in purified FL B-cells compared with reactive B-cells. Only 2 autophagy machinery genes were up-regulated in DLBCL B-cells. In unpurified tissue biopsies, 20 of 46 genes in FL and 2 of 5 genes in DLBCL with increased expression were autophagy machinery genes. Expression of autophagy substrates p62 and LC3 were determined by TMAs. FL samples showed significantly decreased levels of both p62 and LC3 compared with reactive and DLBCL, indicative of an increased autophagy activity in FL. In summary, these results demonstrate that FL showed increased basal autophagy activity, regardless of overexpression of BCL-2 in this disease.

## INTRODUCTION

Macroautophagy (hereafter referred to as autophagy) is a physical and pathological process that allows eukaryotic cells sequester portions of cytoplasm to form autophagosomes and target them for degradation through the fusion of autophagosomes with lysosomes where they are degraded and recycled [1-4]. Growing evidence demonstrates that autophagy plays important and paradoxical roles in tumorigenesis and in the treatment of cancer [4-7]. Autophagy can suppress tumorigenesis by removing damaged organelles/proteins and limiting cell growth and genomic instability [6]. In contrast, induction of autophagy by metabolic stress in apoptosis deficient tumor cells can support tumor cell survival [8]. Abundant preclinical evidence indicates that stress-induced autophagy in tumor cells is predominantly cytoprotective and inhibition of autophagy can enhance tumor cell death by diverse anticancer therapies [9-11].

Follicular lymphoma (FL) is the second most common lymphoma diagnosed in the United States and Western Europe, accounting for approximately 20% of all non-Hodgkin lymphomas (NHLs) and 70% of indolent lymphomas and is generally considered incurable [12, 13]. The t(14;18)(q32;q21) translocation characterizes approximately 85% of FL and 20% of diffuse large B-cell lymphoma (DLBCL) and results in constitutive overexpression of the anti-apoptotic protein BCL-2 [14, 15]. Overexpression of BCL-2 in NHLs plays important roles in disease pathogenesis and resistance to apoptosis. It is currently unknown whether BCL-2 plays an important role in regulation of autophagy in indolent FL and more aggressive DLBCL.

The role of the anti-apoptotic protein BCL-2 in autophagy is currently debated and remains unclear. BCL-2 has been proposed as a major binding partner of Beclin-1 which it binds in a nutrient-dependent manner, consequently down-regulating levels of starvation-mediated autophagy [16]. Recently, it was reported that the anti-apoptotic BCL-2 family members do not directly inhibit components of the autophagic pathway but instead affect autophagy indirectly, owing to their inhibition of Bax and Bak [17]. Autophagy is often activated as an adaptive response against ER stress [18, 19]. In cancer cells, metabolic stress strongly induces autophagy which is sustained when apoptosis is blocked [8, 20, 21]. Questions raised over the roles of BCL-2 in autophagy are: whether overexpression of BCL-2 in human tumors inhibits both apoptosis and autophagy; and whether inhibition of apoptosis by overexpression of BCL-2 could activate the autophagic pathway in favor of prolonged tumor cell survival.

The role of BCL-2 in autophagy in human NHLs has not previously been reported. We hypothesize that overexpression of BCL-2 in FL may cause an increased autophagy activity due to suppression of apoptosis. In this article, we aimed to determine whether overexpression of BCL-2 could alter autophagy status in BCL-2 positive (BCL-2^+^) and negative (BCL-2^−^) DLBCL cell lines, primary FL, DLBCL and reactive (RA) samples using both autophagy RT^2^ Profiler PCR Array and tissue microarray (TMA). We demonstrate for the first time that overexpression of BCL-2 does not inhibit autophagy in human FL.

## RESULTS

### BCL-2^+^ DLBCL cells showed an increased basal autophagy activity

**Figure 1:**
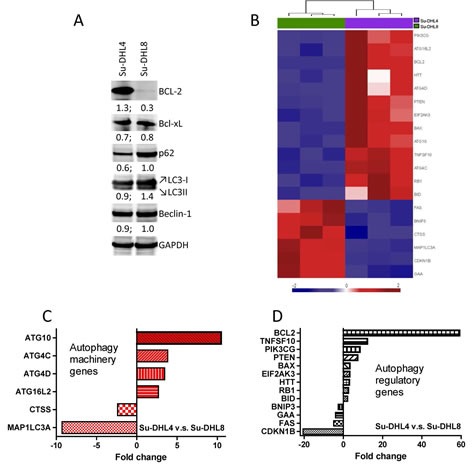
Determination of basal autophagy status in BCL-2 Su-DHL4 and BCL-2 Su-DHL8 cell lines (A) Comparison of autophagy-related protein expression by Western blotting. 50 μg proteins were loaded onto each lane of a 12-well SDS-PAGE gel. Proteins were transferred to a PVDF membrane which was probed with primary antibody at 4°C overnight. Primary antibodies were used at a 1:2000 dilution for GAPDH and at 1:1000 for all other antibodies. Levels of protein expression were measured by densitometry. Numbers below panels of Western blots indicate the ratio of a specific protein to GAPDH. (B) Supervised hierarchical clustering of significantly differentially expressed autophagy machinery and autophagy regulation genes. Heat-map shows triplicate RQ values for Su-DHL4 and Su-DHL8 cell lines. Each column represents an mRNA/RQ value and each row a gene. Gene expression levels are represented as a gradient of blue to red color indicating low and high expression respectively. Side bars were removed for clarity. (C and D) RQ values of significantly increased or decreased autophagy machinery (C) and autophagy regulatory (D) genes differentially expressed in the Su-DHL4 cell line compared with the Su-DHL8 cell line, analyzed using student *t*-test (P<0.05) and represented as fold changes.

To evaluate the impact of BCL-2 overexpression on autophagy in human lymphoma, we first compared the autophagy status of the BCL-2^+^ Su-DHL4 with the BCL-2^−^ Su-DHL8 DLBCL cell lines using Western blotting and the RT^2^ Profiler PCR array (Figure 1). Overexpression of BCL-2 in Su-DHL4 cells was confirmed by Western blotting and there was no differential expression of Beclin-1 or Bcl-xL, another binding partner of Beclin-1, between these two cell lines. SQSTM1/p62 (p62) serves as a link between LC3 and ubiquitinated substrates resulting in these two proteins being incorporated into the completed autophagosome and degraded in the autolysosome [22, 23]. Both p62 and LC3-II showed increased expression in the Su-DHL8 cell line (Figure 1 A), indicative of inhibited autophagic degradation [24], suggesting that the autophagy flux may be inactive in this BCL-2- cell line compared with the BCL-2^+^ Su-DHL4 cells. Using autophagy PCR array, we detected that the Su-DHL4 cell line was also distinguishable from Su-DHL8 cells based on its autophagy-related gene expression profile (GEP) (Figure 1 B and Suppl Figure 1). Four autophagy machinery and nine autophagy regulatory genes were up-regulated in the Su-DHL4 cell line compared to Su-DHL8 cells (Figure 1 C and D). These data support that BCL-2^+^ Su-DHL4 cells may have higher basal level autophagy activity compared with BCL-2^−^ Su-DHL8 cells.

**Figure 2:**
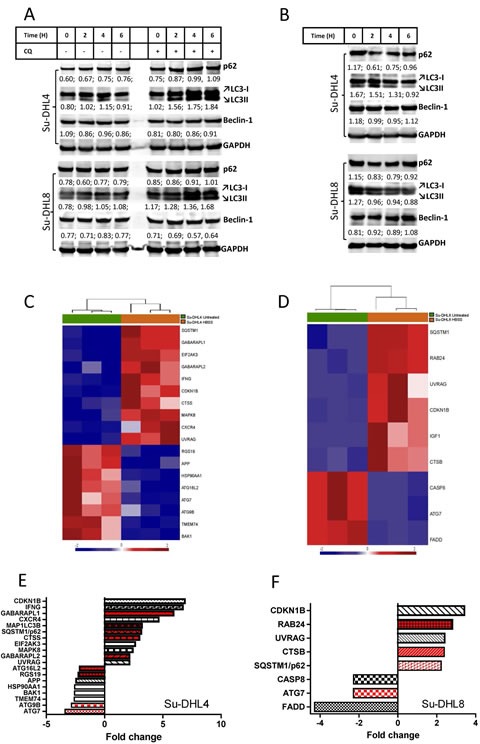
Inhibition or induction of the autophagic flux in Su-DHL4 and Su-DHL8 cell lines (A) Blocking autophagic flux. Cells were incubated in normal culture medium in the presence or absence of 50 μM CQ. (B) Induction of autophagy by starvation. Cells were incubated in HBSS for up to 6 hours. Cells were collected at each indicated time point for protein extraction and Western blotting. Numbers below each band indicate ratios of specific proteins to GAPDH which were determined by densitometry. (C and D) Supervised hierarchical clustering of significantly differentially expressed autophagy related genes in Su-DHL4 (C) and Su-DHL8 (D) cells after incubation in HBSS for 6 hours. Heat-map shows triplicate RQ values for normal and HBSS cultured Su-DHL4 and Su-DHL8 cell lines. Each column represents an mRNA/RQ value and each row a gene. Gene expression levels are represented as a gradient of blue to red color indicating low and high expression respectively. (E and F) RQ values of autophagy related genes significantly differentially expressed in Su-DHL4 (E) and Su-DHL8 (F) cells after incubation in HBSS for 6 hours were analyzed by the paired student *t*-test (p<0.05) and represented as fold changes. Red bars indicate changes in autophagy machinery genes and black and white bars indicate changes in autophagy regulatory genes.

We next sought to determine the capacity of the autophagic flux in both BCL-2^+^ and BCL-2^−^ cells. Inhibition of the autophagic flux by treatment of cells with CQ [7, 25] or induction of autophagy by nutrient deprivation led to increased or decreased p62 and LC3B-I/LC3B-II protein expression respectively in both cell lines (Figure 2 A and B), demonstrating that autophagy activity was not suppressed in the BCL-2^+^ Su-DHL4 cells. Beclin-1 expression did not change in response to either treatment, indicating that its levels are not governed by the autophagy flux. Both Su-DHL4 (Figure 2 C and E and Suppl Figure 2) and Su-DHL8 (Figure 2 D and F and Suppl Figure 3) cell lines responded to nutrient deprivation by up-regulating and down-regulating autophagy-related genes. Among them, *SQSTM1* (p62) and *CDKN1B* genes showed significantly increased expression in both cell lines (Figure 2 E and F). Between 2 and 6 hours starvation, we also noted increased p62 protein expression despite increased autophagic degradation (Figure 2 B). A similar phenomenon has also been observed in mouse embryonic fibroblasts [26]. Increased expression of *CDKN1B* in response to starvation may cause cell cycle arrest in the G1 phase [27]. In addition, the Su-DHL4 cell line also showed significantly increased expression of key autophagy machinery genes including *GABARAPL1*, *GABARAPL2*, *MAP1LC3B* (LC3B) and *CTSS* (Figure 2 C and E). These results suggest that BCL-2^+^ cells may have increased autophagy activity in response to autophagy stress compared with BCL-2^−^ cells.

### FL B-cells showed an increased expression of autophagy-related genes

**Figure 3:**
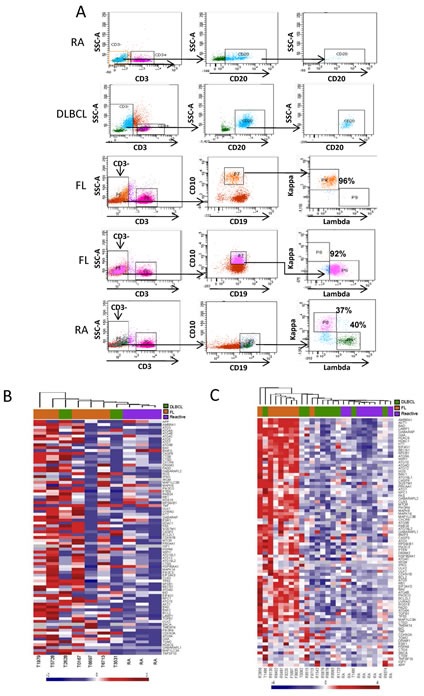
Determination of expression of autophagy related genes in purified and unpurified FL and DLBCL samples (A) Flow sorting of B-cells. Immunophenotyping was used to isolate the B-cell population from human RA, FL and DLBCL single cell suspensions. B-cells were identified as CD3^−^ CD20^+^ for RA and DLBCL samples and CD3^−^/CD10^+^/CD19^+^ for FL samples. Purified FL B-cells were confirmed as being either κ or λ light chain restricted. (B and C) Unsupervised hierarchical clustering using autophagy-related genes expressed in purified B cells (B) and unpurified bulk biopsies (C). Five FL, 2 DLBCL and 3 RA purified samples (B) and 8 FL, 10 DLBCL and 8 RA tissue biopsies (C) were analyzed by qRT-PCR. Heat-map shows RQ values where each column represents a patient and each row a gene. Gene expression levels are represented as a gradient of blue to red color indicating low and high expression respectively; gray indicates missing data.

We next evaluated autophagy gene expression levels in primary FL and DLBCL samples and compared them to RA controls. In order to differentiate autophagy activity in lymphoma B-cells from surrounding stromal cells, tumor-infiltrating T-cells and macrophages, B-cell subsets were isolated by flow cytometry. CD3^+^ T-cells were excluded and CD10^+^/CD19^+^ B-cells (FL) and CD20^+^ B-cells (DLBCL and RA) were purified from primary single cell suspensions. B-cell receptor (BCR) isotype restriction is a hallmark of FL cells, and purified CD19^+^/CD10^+^ FL B-cells were found to be either κ or λ light-chain restricted (Figure 3 A). After flow sorting, mean purities of B-cells were ≥95% for all samples.

We analyzed the autophagy-related GEP of highly purified and unpurified FL and DLBCL diagnostic tissue biopsies and compared them with non-malignant RA samples. The results of unsupervised hierarchical clustering are shown for purified reactive and malignant B-cells (Figure 3 B) and unpurified tissue biopsies (Figure 3 C). Seven and two autophagy machinery genes were up-regulated in purified FL and DLBCL samples, respectively (Table 1), one of which, *MAP1LC3A* was commonly up-regulated in both FL and DLBCL purified B-cells. Only one gene, BNIP3, showed significantly decreased expression in both FL and DLBCL B-cells. BNIP3 is a hypoxia-dependent autophagy inducer and its expression is suppressed in many types of cancer [28] but overexpressed in lung and breast carcinomas [29]. Gene expression patterns in both FL and DLBCL were not associated with Ann Arbor stage or international prognostic index (IPI) scores (data not shown). Among the 46 genes which showed increased expression in these samples, 19 genes in FL and 2 genes in DLBCL were autophagy machinery genes (Figure 3 C and Table 1) and 27 genes in FL and 3 in DLBCL were autophagy regulatory genes. Both *BECN1* and *BCL2* genes were up-regulated in FL but not in DLBCL tissue biopsies. Expression of two lysosomal components *CTSD* (cathepsin D) and *DRAM1* (damage-regulated autophagy modulator 1) [30] was significantly up-regulated in both FL and DLBCL tissue biopsies, suggesting they may be expressed at higher levels in the tumor microenvironment. *CDKN2A* (p16), a tumor-suppressor gene, is up-regulated in both purified and unpurified FL and DLBCL samples (Table 1). To consolidate these findings, increased expression of *BECN*, *MAP1LC3A*, *ATG4B*, *DRAM1* and *CTSD* was validated in unpurified tissue using qRT-PCR. Results were comparable to those obtained from the PCR array (Suppl Table 8). These data demonstrate that both FL and DLBCL samples aberrantly express autophagy genes at the basal levels. In particular, FL samples which frequently overexpress BCL-2 have increased expression of numerous autophagy machinery and regulatory genes.

**Table 1:** Aberrantly expressed autophagy-related genes in purified and unpurified FL and DLBCL samples

	Gene name and function	FL				DLBCL			
		F.C. (UP)	P value	F.C. (P)	P value	F.C. (UP)	P value	F.C. (P)	P value
AUTOPHAGY MACHINERY GENES	BECN1 (1)	2.07	<0.005						
	RGS19 (1)	2.14	<0.05						
	AMBRA1 (1)	2.23	<0.005						
	ATG16L1 (1,3)	2.42	<0.05	3.00	0.24				
	MAP1LC3A (1)	2.60	<0.005	5.38	0.2			5.22	0.33
	GABARAPL1 (1,3)			7.98	0.65				
	GABARAPL2 (1,3)	2.64	<0.005						
	GABARAP (1,2,4)	2.06	<0.005						
	ATG9A (1,3)	3.26	<0.005	2.56	<0.05				
	ATG4D (1,2,3,6)	3.75	<0.005						
	ATG4B (1,2,3,6)	4.66	<0.005						
	ULK1 (1)			3.29	0.06				
	WIPI1 (1)	3.97	<0.005						
	ATG16L2 (3)	2.047	<0.05						
	RAB24 (3)	2.38	<0.05						
	ATG7 (3,5)	2.39	<0.005						
	ATG10 (3)	7.21	<0.005						
	DRAM1 (4)	2.38	<0.05			4.24	<0.05	10.77	0.27
	CTSD (4,7,10)	2.41	<0.005			4.14	<0.05		
	LAMP1 (4,9)	2.65	<0.05	2.02	<0.05				
	HDAC6 (5)	3.91	<0.005	2.23	<0.05				
AUTOPHAGY REGULATORY	BNIP3 (7)			−2.8	0.05			−15.37	0.12
	FADD (7)	2.07	<0.005						
	BAD (7)	2.12	<0.005						
	BID (7)							3.96	0.11
	FAS (7)	2.16	<0.05	3.58	0.10			7.16	0.08
	MTOR (7)	2.18	<0.005						
	MAPK8 (7)	2.31	<0.05						
	BAK1 (7)	2.31	<0.05						
	CLN3 (7)	2.33	<0.005						
	CXCR4 (7)	2.25	<0.05						
	AKT1 (7)	2.69	<0.005						
	BAX (7)	2.95	<0.05						
	IFNG (7)			3.04	0.34	6.54	0.1	−5	0.44
	HTT (7)	3.20	<0.005						
	HDAC1 (7)	3.23	<0.005					−2.0	<0.05
	PIK3CG (7)	3.33	<0.005					−8.07	<0.005
	BCL2L1	3.81	<0.005						
	DAPK1 (7)	5.04	<0.05						
	BCL2 (7)	8.88	<0.005	7.03	0.1				
GENES	TGM2 (7)					2.99	<0.05	4.56	0.16
	TP53 (7,8)	2.98	<0.005						
	CDKN1B (7,8)	2.11	<0.05						
	CDKN2A (7,8)	6.19	<0.05	4.22	0.11	3.27	0.12	4.12	<0.05
	EIF4G1 (10)	2.16	<0.005						
	UVRAG (10)	2.33	<0.005						
	RPS6KB1 (10)	2.36	<0.005						
	MAPK14 (10)	2.50	<0.05						
	ESR1 (10)	2.52	<0.05						
	GAA (10)	2.95	<0.005						
	TMEM74 (10)	3.56	0.31	3.71	0.51			5.69	0.33
	HGS (10)	7.38	<0.05						

**Fold changes (F.C.)** in gene expression levels of unpurified (UP) FL or DLBCL samples were compared with unpurified RA-LNs and those of purified (P) FL or DLBCL samples were compared with purified reactive B cells. Genes listed in the table are genes with expression changes greater than or less than 3 fold and those greater than or less than 2 fold with a P value <0.05. Numbers of samples: purified RA n=3; FL n=5; DLBCL n=2; unpurified RA n=8; FL=8; DLBCL n=10. Significantly increased or decreased fold changes were identified using a Mann Whitney *U* test. Numbers in the first column indicate the functions of these genes, according to the classification of human autophagy PCR array (Sabiosciences): (1-6) Autophagy machinery genes: (1) Genes involved in autophagic vacuole formation; (2) Gene responsible for protein targeting to membrane/vacuole; (3) Genes responsible for protein transport; (4) Genes linking autophagosome to lysosome; (5) Genes involved in protein ubiquitination; (6) Genes with protease activity; (7-10) Autophagy regulatory genes: (7) Co-regulators of autophagy and apoptosis; (8) Co-regulators of autophagy and the cell cycle; (9) Autophagy induction by intracellular pathogens; (10) Autophagy in responsible to other intracellular signals.

In the cohort of purified samples, two FL patients (T1979 and T5728) with high global expression of autophagy genes subsequently underwent transformation to the more aggressive DLBCL later in their clinical course (Suppl Figure 4 A and Suppl Table 1). In unpurified tissue biopsies, 7 of 8 FL and 1 of 10 DLBCL samples showed increased global expression of autophagy genes compared to RA tissues (Suppl Figure 4 B). A larger number of purified DLBCL samples will be required to establish aberrant expression of autophagy genes in this disease.

### FL showed significantly decreased expression of autophagy substrates p62 and LC3 proteins

**Figure 4:**
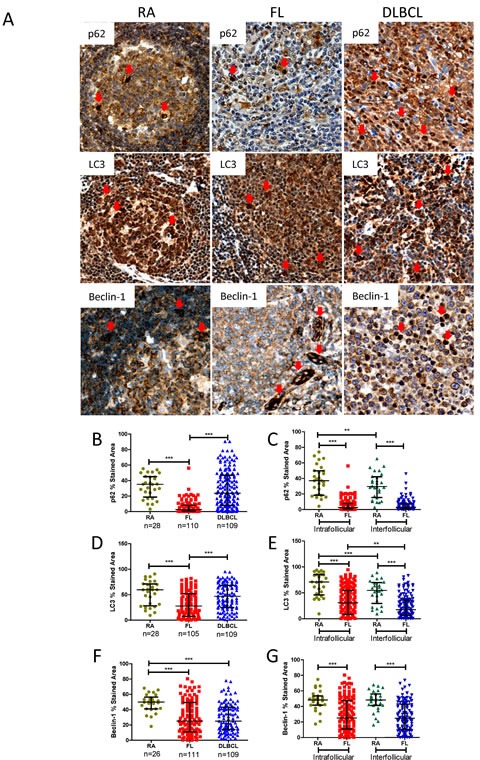
Comparison of p62, LC3 and Beclin-1 protein expression in FL and DLBCL with RA-LN (A) Representative histochemical stained images of p62, LC3, and Beclin-1 in RA, FL and DLBCL. Proteins were stained with polyclonal anti-p62, polyclonal anti-LC3B antibody, or a mouse anti-Beclin-1 antibody respectively. Detailed information of antibodies and their dilution are listed in the Suppl Table 5. Red arrows indicate positive cells. (B-G) Statistical analysis p62 (B and C), LC3B (D and E) and Beclin-1 (F and G) expression. All data presented are medians with interquartile ranges. Sample numbers for RA and FL in C, E and G are as same as listed in B, D and F. Statistical difference between samples was analyzed by unpaired Mann-Whitney *U* test. *P<0.05, **P<0.01, and ***P<0.0001.

To establish the autophagy status of FL and DLBCL, primary FL, DLBCL and RA LN tissue biopsies on TMAs were stained using IHC for the autophagy substrate proteins p62 and LC3, and autophagy initiating protein Beclin-1 (Figure 4A). RA and FL samples were stained with CD10 antibody to distinguish follicular center B-cells from surrounding cells. CD10^+^ area were classified as intra-follicular areas; CD10^−^ areas were classified as inter-follicular areas [31] (Suppl Figure 5A).

First, protein expression in FL intra-follicular areas was compared with RA and DLBCL whole core samples. p62, LC3 and Beclin-1 showed significantly decreased expression in FL intra-follicular areas compared with DLBCL and RA controls. Only Beclin-1 displayed significantly decreased expression in DLBCL (Figure 4 B, D and F). Most strikingly, all FL samples showed consistently lower levels of p62. Expression of p62 in DLBCL and LC3 and Beclin-1 in FL and DLBCL displayed a heterogeneous expression pattern. In FL, significantly decreased expression of p62, LC3 and Beclin-1 was observed in both intra-follicular and non-malignant inter-follicular areas (Figure 4 C, E and G), suggesting autophagy may be altered in both malignant FL cells and surrounding tumor infiltrating cells. Approximately 91% FL samples were BCL-2 positive (>30% of stained area); DLBCL samples displayed a heterogeneous BCL-2 expression pattern (Suppl Figure 5 B and C). These results demonstrate that FL has an increased basal autophagy activity, regardless of overexpression of BCL-2.

**Figure 5:**
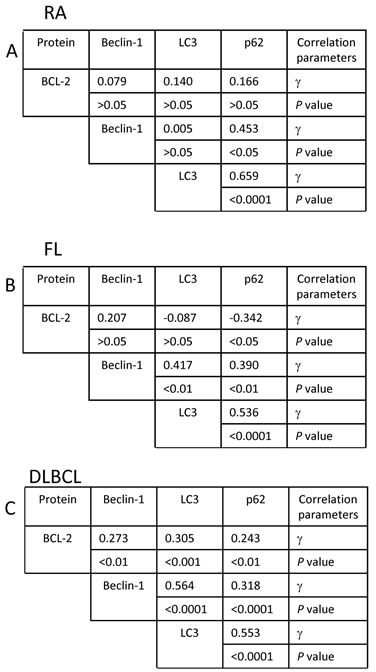
Multiple analysis of correlation between BCL-2, p62, LC3 and Beclin-1 (A) RA; (B) FL and (C) DLBCL. Correlation was analyzed by Pearson product-moment correlation coefficient test. ‘γ’ indicates correlation coefficient. Sample numbers, RA=30; FL=50 and DLBCL=109.

In order to understand the association between BCL-2 and p62, LC3 or Beclin-1, correlations between these proteins in RA, FL and DLBCL were analyzed by Pearson product-moment correlation method (Figure 5). BCL-2 levels showed a negative correlation with p62 in FL (P<0.05) and positive correlation with p62, LC3 or Beclin-1 in DLBCL (P<0.01), suggesting that BCL-2 does not play a dominant role in autophagy status in these lymphomas. Expression levels of p62 and LC3 showed strong positive correlation (P<0.0001) in RA, FL and DLBCL samples, indicating that p62 or LC3, individually or in combination, can be used as a marker for evaluating autophagy activity. Interestingly, Beclin-1 expression levels were positively correlated with levels of p62 and LC3 in both FL (P<0.01) and DLBCL (P <0.0001), suggesting that Beclin-1 levels is not positively associated with autophagy activity.

### Cathepsin D and tissue transglutaminase-2 are highly expressed in the tissue macrophages

Gene expression profiling of DLBCL malignant B-cells may be affected by the presence of stromal cells [32]. *CTSD* showed significantly increased expression at the gene level in FL and DLBCL sample biopsies but not in purified B-cells. Increased expression of *TGM2* (Tissue transglutaminase-2) was detected in both purified and unpurified DLBCL samples (Table 1). To distinguish the origins of these components, we evaluated expression of cathepsin D and TGM2 proteins in previously un-treated FL, DLBCL and RA tissue biopsies and the results were reviewed by expert histopathologist (MC and RC). The expression pattern of cathepsin D and TGM2 suggested that they are not expressed by lymphoma cells, but rather macrophages (Figure 5 A). To confirm cells highly expressing cathepsin D and/or TGM2 are tumor infiltrating cells, DLBCL and RA TMAs were stained with CD68, a marker for tumor-associated macrophages (TAMs) [33, 34] (Figure 5 B). These data confirmed that cells expressing cathepsin D and/or TGM2 are indeed macrophages, although co-localization between these proteins and CD68 was not evaluated. Cathepsin D expression was significantly lower in FL but significantly higher in DLBCL samples compared with RA controls (Figure 5 C), while both FL and DLBCL samples showed decreased expression of TGM2 (Figure 5 D). Neither cathepsin D nor TGM2 expression was correlated with autophagy status in FL or DLBCL (data not shown). Both cathepsin D and TGM2 showed strong positive correlation (P<0.0001) with CD68 (Supp Figure 6 A and B). Expression of cathepsin D and TGM2 were also strongly correlated (P<0.0001) (Supp Figure 6 C). These results suggest that higher expression of autophagy genes in TAMs may lead to misinterpretation of the gene signature of malignant B-cells when analyzing autophagy in unpurified tissue biopsies by PCR array.

**Figure 6:**
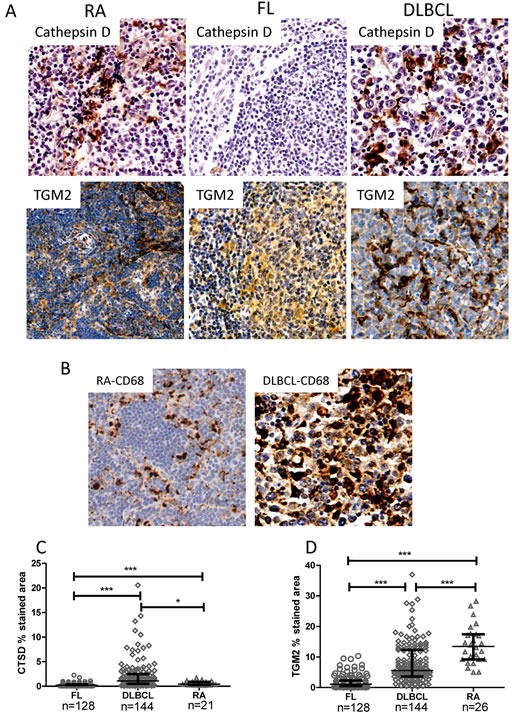
Immuno-histochemical staining of cathepsin D, TGM2 and CD68 (A) Representative images of cathepsin D and TGM2 expression in RA, FL and DLBCL. (B) Representative images of CD68 expression in RA and DLBCL. Antibody dilutions were 1:1000, 1:750, or 1:8000 for anti-cathepsin D, anti-TGM2, or anti-CD68 antibodies, respectively. Images were taken with a Leixa DM2500 microscope: original magnification X200. Cathepsin D, TGM2 and CD68 protein expression levels were defined as % stained viable tissue area. (C and D) Statistical analysis of protein levels of cathepsin D (B) and TGM2 (C) were calculated as the percent stained area of viable tissue. Data collected for RA and DLBCL were from whole cores and for FL were from the CD10^+^ intra-follicular area. Significantly increased or decreased expression between samples was analyzed by the Mann-Whitney *U* test. Numbers of samples used for analysis were indicated.

## DISCUSSION

Here we report that FL, an indolent NHL which frequently overexpresses the anti-apoptotic protein BCL-2, showed significantly increased expression of key autophagy genes and decreased levels of autophagy substrate protein p62 and LC3 compared with RA B-cell controls. Whereas, the autophagy-related GEP and levels of autophagy substrate p62 and LC3 proteins in DLBCL, an aggressive NHL, was more similar to those of RA B-cell controls. A constitutive basal level of autophagy in normal tissue provides an important homeostatic, housekeeping function to survive stress, such as nutrient deprivation [21, 35]. The role of autophagy in cancer is complex as it can prevent tumor initiation by suppressing chronic tissue damage, inflammation, and genome instability via its quality control function or it can sustain tumor metabolism, growth, and survival via nutrient recycling [36], suggesting a link between dysregulated autophagy and tumor progression.

Higher expression of the anti-apoptotic protein BCL-2 is more common in B-cell NHL than T-cell NHL and is heterogeneously expressed among the different histological subtypes [37]. Although BCL-2 is a well-established anti-apoptotic protein, it was also proposed to suppress autophagy by binding and inhibiting Beclin-1 in a cardiac BCL-2 transgenic mice model [16]. A recent finding demonstrated that BCL-2 does not bind directly to Beclin-1 but instead regulate autophagy by inhibiting Bax/Bak mediated apoptosis [17]. We therefore determined the role of BCL-2 on autophagy in BCL-2^+^ and BCL-2^−^ cell lines and human primary lymphoma samples. Higher levels of autophagy machinery genes were found in BCL-2^+^ cells compared with the BCL-2^−^ cell line. BCL-2^+^ and BCL-2^−^ DLBCL cell lines showed similar autophagy flux activity upon either autophagy inhibition by CQ or autophagy induction by nutrient-deprivation, evidenced by accumulation or degradation of p62 and LC3 proteins, respectively. Interestingly, the BCL-2^+^ cell line up-regulated more autophagy-related genes in response to starvation compared to BCL-2^−^ DLBCL cells. These results demonstrate that the autophagic flux is not altered by the BCL-2 protein in human malignant B-cells. In fact, BCL-2^+^ cells up-regulate more autophagy-related genes than BCL-2^−^ DLBCL cells in both the resting state and in response to autophagy induction.

As expected, the BCL-2 gene was highly expressed in both purified FL B-cells and unpurified tissue biopsies compared with RA and DLBCL primary samples. To identify expression signatures of autophagy-associated genes in the malignant population specifically, B-cells were purified from FL and DLBCL tissue biopsies by flow sorting. Normal B-cells were also purified from RA-LNs which served as controls. Seven autophagy machinery genes were up-regulated in purified FL B-cells, including *ATG9A, ATG16L1, MAP1LC3A, GABARAPL1* and *ULK1*, which are involved in autophagosome formation and protein transport; *LAMP1*, involved in autolysosome formation; and *HDAC6*, involved in protein ubiquitination. This result demonstrates that FL B-cells, which overexpress BCL-2 up-regulate the basal autophagy activity at the gene level. We have also confirmed by decreased expression of autophagy substrate proteins p62 and LC3 in FL tissue specimens using TMAs and IHC, demonstrating an active autophagy at the protein levels in FL. Nevertheless, there is no strong and clear correlation between BCL-2 expression levels and autophagy activity. We therefore propose that autophagy activity in lymphoma samples may not be controlled by BCL-2.

FL frequently transforms to the more aggressive DLBCL. Our data showed that only one gene, *MAP1LC3A,* was up-regulated in DLBCL B-cells. It was recently reported that autophagy is positively regulated by *LITAF* which is silenced by promoter hypermethylation in germinal center-derived B-cell lymphomas, suggesting that autophagy may be inhibited in these lymphomas [38]. We found that levels of p62 and LC3 proteins showed a heterogeneous expression pattern in DLBCL and had no significant difference compared with RA controls. In addition, expression levels of p62 and LC3 did not show association with clinical outcomes of patients with FL (data not shown). This suggests that up-regulated autophagy in FL may not be associated with transformation to DLBCL. Instead, active autophagy in FL may even suppress tumor progression by eliminating damaged organelles and controlling genetic instability.

Increased expression of autophagy-related genes was more readily detected in FL bulk tissue biopsies. Similar to FL purified B-cells, FL unpurified tissue also showed up-regulation of autophagy machinery genes, including *ATG16L1*, *MAP1LC3A*, *ATG9*, *LAMP1* and *HDAC6*; many other autophagy machinery and regulatory genes were also significantly up-regulated. Among the up-regulated autophagy regulatory genes, *TP53*, *MAPK8*, *HDAC1*, *DAPK1*, *CDKN1B*, *CDKN2A*, *UVRAG*, and *RPS6KB1* are positive regulators of autophagy, whereas *AKT1*, *PIK3CG*, *BCL-2*, *BCL-2L1*, *mTOR*, *EIF4G1*, and *MAPK14* are negative autophagy regulators. It is obvious that more aberrantly expressed autophagy-related genes were detected in unpurified FL samples compared with purified B-cells. Our previous studies show that FL tissues have increased numbers of CD163^+^ infiltrating macrophages [39] and CD4^+^ T-cells [40] in the microenvironment. This suggests that autophagy activity might also be altered in FL tumor infiltrating cells and this is being actively explored. Notably, we did not find evidence of upregulation of autophagy related genes in our previous studies of GEP of the tumor infiltrating T-cells in FL [31].

Fewer autophagy-related genes had altered expression levels in DLBCL, regardless of sample purification. Significantly up-regulated genes in DLBCL samples included *CTSD*, *DRAM1* and *TGM2*. *CTSD* and *DRAM1* are lysosomal proteins which regulate the autophagic flux through the lysosome [30, 41], while *TGM2* is involved in autophagy-dependent clearance of ubiquitinated proteins [42]. We therefore tested the origin of cells expressing high levels of cathepsin D and TGM2 using TMAs. Expression of both cathepsin D and TGM2 proteins was significantly lower in FL samples but significantly higher in DLBCL samples compared with RA controls. Morphological features of cells expressing higher cathepsin D or TGM2 were identical to CD68-expressing TAMs. Indeed, expression levels of both cathepsin D and TGM2 were strongly correlated to CD68 expression levels in DLBCL. We found that increased cathepsin D expression was associated with a shorter overall survival of DLBCL patients (data not shown), in agreement with a previous report by Nicotra et al [43]. TGM2 has been reported as a marker for progression and therapeutic intervention in colorectal cancer and non-small cell lung cancer.[44, 45] However, the role of TGM2 in DLBCL is unknown. We found that TGM2 expression is not associated with shorter overall survival or other prognostic markers in DLBCL (data not shown). Nevertheless, *CTSD* and *TGM2* expression levels in DLBCL did not reflect their expression in malignant B-cells. We therefore propose that determination of autophagy-related gene expression using unpurified lymphoma specimens could be distorted by high lysosome-containing TAMs.

In summary, the role of BCL-2 in autophagy is currently elusive. Using the RT^2^ Profiler Human Autophagy PCR array and TMAs, we demonstrate that basal autophagy activity was up-regulated in primary BCL-2 overexpressing FL B-cells and their microenviromental cells. A greater number of autophagy machinery genes were up-regulated in the BCL-2^+^ DLBCL cell line Su-DHL4 at the basal level and in response to stress, indicating that overexpression of BCL-2 does not inhibit the autophagic flux. Instead, inhibition of apoptosis by overexpression of BCL-2 may switch on autophagy in the cell in favor of eliminating aged organelles and damaged proteins. However, the mechanism by which FL cells up-regulate autophagy-related gene expression is not clear and may be due to multiple factors. We therefore propose that overexpression of the anti-apoptotic protein BCL-2 does not suppress autophagy activity in human FL.

## MATERIALS AND METHODS

### Cell lines and cell culture

Human DLBCL cell lines Su-DHL4 (BCL-2^+^) and Su-DHL8 (BCL-2^−^) [46] were used in this study. Cells were cultured in RPMI-1640 medium supplemented with 10% heat-inactivated fetal calf serum (FCS), 25mM HEPES, and 2.0mM L-glutamine at 37°C in a 5% CO_2_ humidified incubator. To induce autophagy, cells were cultured in HBSS for up to 6 hours.

### Human samples and ethical considerations

Ethical approval for the human biological materials used in this study was obtained in accordance with the requirements of the East London and the City Health Authority Local Research Ethics Committee (Ref. No. 10/H0704/65). All samples were obtained from patients by informed consent. Patients selected presented at St. Bartholomew's hospital between the years 1970-2012. For RT^2^ Profiler PCR array analysis, lymph node (LN) biopsies and cryopreserved single cell suspensions were obtained from diagnostic, previously un-treated FL (n=13) and DLBCL (n=11) patients (Suppl Table 1 and 2); RA-LNs (n=11) were used as controls. For tissue microarrays (TMAs), LN biopsies from 128 FL and 144 DLBCL patients at diagnosis for whom quality formalin fixed paraffin embedded tissue, clinical and follow-up data were available, as well as 28 reactive LN biopsies, were included on the TMAs (Suppl Table 3 and 4).

### Reagents

RT^2^ Profiler Human Autophagy PCR Array (PAHS084ZE), RNeasy mini kit, RT^2^ First Strand Kit, primers for qPCR validation, including *BECN* (PPH05670B), *MAP1LC3A* (PPH19436A), *ATG4B* (PPH15916A), *DRAM1* (PPH19768F), *CTSD* (PPH00112F) and *RPLPO* (PPH21138F) were purchased from Qiagen-Sabiosciences. 3,3′-diaminobenzidine (DAB) was from BioGenex. TRIzol® reagent, 4-12% NuPAGE gels and Hanks balanced salt solution (HBSS) were from Invitrogen. Chloroquine (CQ), 4′,6-diamidino-2-phenylindole (DAPI), and all other chemicals used were from Sigma. Antibodies used in this study are listed in Suppl Table 5.

### Purification of B-cells by flow cytometry sorting

Primary single cell suspensions (10^6^cells/ml) were washed once with washing buffer containing 2% FCS in PBS (phosphate buffered saline). Non-specific bindings were blocked by incubating cells with 2% human anti-γ-globulin antibody for 30 min at 4°C. Cells were stained with conjugated anti-CD3/anti-CD20 or anti-CD3/anti-CD19/anti-CD10 antibodies for 30 min at 4°C and subsequently washed once with washing buffer. Cells were resuspended in blocking buffer containing DAPI (50ng/ml) and sorted on a BD FACSAria II Cell Sorter. DAPI was used to discriminate live and dead cells. Following selection of DAPI negative cells, T-cells were excluded by gating on CD3 negative (CD3^−^) cells. B-cells were isolated from the DAPI^−^/CD3^−^ population based on expression of B-cell markers. DLBCL and reactive B-cells were identified by CD20 expression while FL B-cells were isolated based on dual expression of CD19 and CD10. FL B-cells were further confirmed as the malignant cell population by demonstrating kappa/lambda (κ/λ) light chain restriction [47].

### RNA extraction and cDNA conversion

Total RNA was isolated from purified single B-cell suspensions or solid LN tissue biopsies with TRIzol® and/or the RNeasy mini kit. RNA quality was assessed using the Agilent 2100 Bioanalyzer (Agilent Technologies) and a Nanodrop spectrophotometer (Thermo-Scientific); all samples had an RNA integrity number (RIN) greater than 6 and 260/280 ratios higher than 1.9. RNA (300ng) was converted to cDNA by RT-PCR using the RT^2^ First Strand Kit.

### RT2 Profiler PCR array for detection of expression of autophagy related genes

The RT^2^ Profiler PCR Array combines qRT-PCR technology with a microarray format to allow the simultaneous detection of multiple gene expression levels in a rapid manner. The RT^2^ Profiler Human Autophagy PCR Array contains primers against 84 genes involved in different stages of the autophagy pathway (Suppl Table 6 and 7) as well as primers against five housekeeping genes which are used for data normalization. According to the manufacturer's protocol, qRT-PCR was performed by adding 2.8ng cDNA mixed with RT^2^ SYBR Green Mastermix (Sabiosiciences) to each well which already contained primers directed against the gene of interest. mRNA levels were analyzed using the ABI Prism 7900HT Fast *Real-Time* PCR System (Applied Biosystems) and a dissociation curve analysis step included to verify PCR specificity. Relative quantity (RQ) values were calculated using the formula RQ = 2^−ΔΔC^_T_. Target cycle threshold (C_T_) values were normalized to the housekeeping gene RPLPO generating a ΔC_T_ value. The average ΔC_T_ of reactive controls was used as the calibrator sample on a per gene basis and was subtracted from each ΔC_T_ generating a delta ΔC_T_ value which was linearized by raising to the power of 2 (2^−ΔΔC^_T_). Genes with a fold change ≥3 or ≤-3 or a FC ≥2 or ≤-2 and a *p* value <0.05 using a Mann-Whitney *U* test or student *t*-test were taken to be biologically meaningful. Hierarchical clustering was performed using Euclidean distance measure and an average agglomeration available within the R statistical computing environment.

### qRT-PCR validation

Following conversion of RNA (500ng) to cDNA, samples were prepared for qRT-PCR as previously described and the qRT-PCR assay run under the previously stipulated conditions. All genes validated, including the housekeeping gene RPLPO, were analyzed in triplicate and a no-template control (NTC) also included per gene. Primers used in validation experiments were identical to those present on the RT^2^ Profiler Human Autophagy PCR Array.

### Tissue microarray (TMAs) and IHC analysis

TMAs were constructed using a semi-automated arraying system (TMABooster-Alphelys). Sections of biopsy material were stained with hemotoxylin and eosin and reviewed by an expert histopathologist (C.M.) who identified areas rich in malignant cells. Triplicate 1mm^2^ cores were then taken from these areas, arrayed and stained as previously described [31, 33, 34]. Slides were digitalized and image analysis was performed using a digital pathology system (Ariol, Lieca Microsystems). The Ariol image analysis classifier was trained based on the hue, saturation and intensity of DAB staining, such that only areas stained above a pre-determined threshold representing the highest intensity staining were classed as positive, and lower level background expression was excluded. A further classifier was used to determine the total viable tissue area. Combining these two classifiers, the percent stained area was calculated. In FL cores, intra-follicular areas were selected based on expression of CD10 and clear follicle morphology. Results were manually and blindly reviewed and reported as an average of the triplicate cores. Protein levels were expressed as % stained area [33].

### Western blotting

Proteins were extracted with lysis buffer and 50 μg of proteins added to each lane of 4-12% NuPAGE gels and Western blotting was performed as previously described [33].

### Statistical analysis

Statistical analysis was performed using GraphPad Prism software (version 5.03). Data are shown as either mean ± SD or median with interquartile range when variation was high. Significant differences between groups with unequal size were analyzed with the Mann-Whitney *U* test and those with equal size were analyzed using the student *t*-test. Pearson product-moment correlation method was used to analyze linear correlation between two groups. All *P*-values less than 0.05 were considered statistically significant.

## SUPPLEMENTARY MATERIAL FIGURES AND TABLES





## References

[1] Jia L, Dourmashkin RR, Allen PD, Gray AB, Newland AC, Kelsey SM (1997). Inhibition of autophagy abrogates tumour necrosis factor alpha induced apoptosis in human T-lymphoblastic leukaemic cells. Br J Haematol.

[2] Mizushima N, Levine B, Cuervo AM, Klionsky DJ (2008). Autophagy fights disease through cellular self-digestion. Nature.

[3] White E (2012). Deconvoluting the context-dependent role for autophagy in cancer. Nat Rev Cancer.

[4] Rao S, Tortola L, Perlot T, Wirnsberger G, Novatchkova M, Nitsch R, Sykacek P, Frank L, Schramek D, Komnenovic V, Sigl V, Aumayr K, Schmauss G, Fellner N, Handschuh S, Glosmann M (2014). A dual role for autophagy in a murine model of lung cancer. Nat Commun.

[5] Wu WK, Coffelt SB, Cho CH, Wang XJ, Lee CW, Chan FK, Yu J, Sung JJ (2012). The autophagic paradox in cancer therapy. Oncogene.

[6] Mathew R, Karp CM, Beaudoin B, Vuong N, Chen G, Chen HY, Bray K, Reddy A, Bhanot G, Gelinas C, Dipaola RS, Karantza-Wadsworth V, White E (2009). Autophagy suppresses tumorigenesis through elimination of p62. Cell.

[7] Jia L, Gopinathan G, Sukumar JT, Gribben JG (2012). Blocking Autophagy Prevents Bortezomib-Induced NF-kappaB Activation by Reducing I-kappaBalpha Degradation in Lymphoma Cells. PLoS One.

[8] Degenhardt K, Mathew R, Beaudoin B, Bray K, Anderson D, Chen G, Mukherjee C, Shi Y, Gelinas C, Fan Y, Nelson DA, Jin S, White E (2006). Autophagy promotes tumor cell survival and restricts necrosis, inflammation, and tumorigenesis. Cancer Cell.

[9] Yang ZJ, Chee CE, Huang S, Sinicrope FA (2011). The role of autophagy in cancer: therapeutic implications. Mol Cancer Ther.

[10] Vogl DT, Stadtmauer EA, Tan KS, Heitjan DF, Davis LE, Pontiggia L, Rangwala R, Piao S, Chang YC, Scott EC, Paul TM, Nichols CW, Porter DL, Kaplan J, Mallon G, Bradner JE (2014). Combined autophagy and proteasome inhibition: A phase 1 trial of hydroxychloroquine and bortezomib in patients with relapsed/refractory myeloma. Autophagy.

[11] Lamoureux F, Thomas C, Crafter C, Kumano M, Zhang F, Davies BR, Gleave ME, Zoubeidi A (2013). Blocked autophagy using lysosomotropic agents sensitizes resistant prostate tumor cells to the novel Akt inhibitor AZD5363. Clin Cancer Res.

[12] Inami Y, Waguri S, Sakamoto A, Kouno T, Nakada K, Hino O, Watanabe S, Ando J, Iwadate M, Yamamoto M, Lee MS, Tanaka K, Komatsu M (2011). Persistent activation of Nrf2 through p62 in hepatocellular carcinoma cells. J Cell Biol.

[13] Isogai S, Morimoto D, Arita K, Unzai S, Tenno T, Hasegawa J, Sou YS, Komatsu M, Tanaka K, Shirakawa M, Tochio H (2011). Crystal structure of the ubiquitin-associated (UBA) domain of p62 and its interaction with ubiquitin. J Biol Chem.

[14] Yunis JJ, Frizzera G, Oken MM, McKenna J, Theologides A, Arnesen M (1987). Multiple recurrent genomic defects in follicular lymphoma. A possible model for cancer. N Engl J Med.

[15] Fukuhara S, Rowley JD, Variakojis D, Golomb HM (1979). Chromosome abnormalities in poorly differentiated lymphocytic lymphoma. Cancer Res.

[16] Pattingre S, Tassa A, Qu X, Garuti R, Liang XH, Mizushima N, Packer M, Schneider MD, Levine B (2005). Bcl-2 antiapoptotic proteins inhibit Beclin 1-dependent autophagy. Cell.

[17] Lindqvist LM, Heinlein M, Huang DC, Vaux DL (2014). Prosurvival Bcl-2 family members affect autophagy only indirectly, by inhibiting Bax and Bak. Proc Natl Acad Sci U S A.

[18] Kuma A, Hatano M, Matsui M, Yamamoto A, Nakaya H, Yoshimori T, Ohsumi Y, Tokuhisa T, Mizushima N (2004). The role of autophagy during the early neonatal starvation period. Nature.

[19] Nikoletopoulou V, Markaki M, Palikaras K, Tavernarakis N (2013). Crosstalk between apoptosis, necrosis and autophagy. Biochim Biophys Acta.

[20] Karantza-Wadsworth V, Patel S, Kravchuk O, Chen G, Mathew R, Jin S, White E (2007). Autophagy mitigates metabolic stress and genome damage in mammary tumorigenesis. Genes Dev.

[21] Mathew R, Karantza-Wadsworth V, White E (2007). Role of autophagy in cancer. Nat Rev Cancer.

[22] Pankiv S, Clausen TH, Lamark T, Brech A, Bruun JA, Outzen H, Overvatn A, Bjorkoy G, Johansen T (2007). p62/SQSTM1 binds directly to Atg8/LC3 to facilitate degradation of ubiquitinated protein aggregates by autophagy. J Biol Chem.

[23] Gratas C, Sery Q, Rabe M, Oliver L, Vallette FM (2014). Bak and Mcl-1 are essential for Temozolomide induced cell death in human glioma. Oncotarget.

[24] Klionsky DJ, Abeliovich H, Agostinis P, Agrawal DK, Aliev G, Askew DS, Baba M, Baehrecke EH, Bahr BA, Ballabio A, Bamber BA, Bassham DC, Bergamini E, Bi X, Biard-Piechaczyk M, Blum JS (2008). Guidelines for the use and interpretation of assays for monitoring autophagy in higher eukaryotes. Autophagy.

[25] Amaravadi RK, Yu D, Lum JJ, Bui T, Christophorou MA, Evan GI, Thomas-Tikhonenko A, Thompson CB (2007). Autophagy inhibition enhances therapy-induced apoptosis in a Myc-induced model of lymphoma. J Clin Invest.

[26] Sahani MH, Itakura E, Mizushima N (2014). Expression of the autophagy substrate SQSTM1/p62 is restored during prolonged starvation depending on transcriptional upregulation and autophagy-derived amino acids. Autophagy.

[27] Kato JY, Matsuoka M, Polyak K, Massague J, Sherr CJ (1994). Cyclic AMP-induced G1 phase arrest mediated by an inhibitor (p27Kip1) of cyclin-dependent kinase 4 activation. Cell.

[28] Band M, Joel A, Hernandez A, Avivi A (2009). Hypoxia-induced BNIP3 expression and mitophagy: in vivo comparison of the rat and the hypoxia-tolerant mole rat, Spalax ehrenbergi. FASEB J.

[29] Vijayalingam S, Pillai SG, Rashmi R, Subramanian T, Sagartz JE, Chinnadurai G (2010). Overexpression of BH3-Only Protein BNIP3 Leads to Enhanced Tumor Growth. Genes Cancer.

[30] Zhang XD, Qi L, Wu JC, Qin ZH (2013). DRAM1 regulates autophagy flux through lysosomes. PLoS One.

[31] Kiaii S, Clear AJ, Ramsay AG, Davies D, Sangaralingam A, Lee A, Calaminici M, Neuberg DS, Gribben JG (2013). Follicular lymphoma cells induce changes in T-cell gene expression and function: potential impact on survival and risk of transformation. J Clin Oncol.

[32] Lenz G, Wright G, Dave SS, Xiao W, Powell J, Zhao H, Xu W, Tan B, Goldschmidt N, Iqbal J, Vose J, Bast M, Fu K, Weisenburger DD, Greiner TC, Armitage JO (2008). Stromal gene signatures in large-B-cell lymphomas. N Engl J Med.

[33] Jia L, Clear A, Liu FT, Matthews J, Uddin N, McCarthy A, Hoxha E, Durance C, Iqbal S, Gribben JG (2014). Extracellular HMGB1 promotes differentiation of nurse-like cells in chronic lymphocytic leukemia. Blood.

[34] Greaves P, Clear A, Coutinho R, Wilson A, Matthews J, Owen A, Shanyinde M, Lister TA, Calaminici M, Gribben JG (2012). Expression of FOXP3, CD68, and CD20 at Diagnosis in the Microenvironment of Classical Hodgkin Lymphoma Is Predictive of Outcome. J Clin Oncol.

[35] Okatsu K, Saisho K, Shimanuki M, Nakada K, Shitara H, Sou YS, Kimura M, Sato S, Hattori N, Komatsu M, Tanaka K, Matsuda N (2010). p62/SQSTM1 cooperates with Parkin for perinuclear clustering of depolarized mitochondria. Genes Cells.

[36] Ichimura Y, Komatsu M (2010). Selective degradation of p62 by autophagy. Semin Immunopathol.

[37] Hermine O, Haioun C, Lepage E, d'Agay MF, Briere J, Lavignac C, Fillet G, Salles G, Marolleau JP, Diebold J, Reyas F, Gaulard P (1996). Prognostic significance of bcl-2 protein expression in aggressive non-Hodgkin's lymphoma. Groupe d'Etude des Lymphomes de l'Adulte (GELA). Blood.

[38] Bertolo C, Roa S, Sagardoy A, Mena-Varas M, Robles EF, Martinez-Ferrandis JI, Sagaert X, Tousseyn T, Orta A, Lossos IS, Amar S, Natkunam Y, Briones J, Melnick A, Malumbres R, Martinez-Climent JA (2013). LITAF, a BCL6 target gene, regulates autophagy in mature B-cell lymphomas. Br J Haematol.

[39] Clear AJ, Lee AM, Calaminici M, Ramsay AG, Morris KJ, Hallam S, Kelly G, Macdougall F, Lister TA, Gribben JG (2010). Increased angiogenic sprouting in poor prognosis FL is associated with elevated numbers of CD163+ macrophages within the immediate sprouting microenvironment. Blood.

[40] Lee AM, Clear AJ, Calaminici M, Davies AJ, Jordan S, MacDougall F, Matthews J, Norton AJ, Gribben JG, Lister TA, Goff LK (2006). Number of CD4+ cells and location of forkhead box protein P3-positive cells in diagnostic follicular lymphoma tissue microarrays correlates with outcome. J Clin Oncol.

[41] Qiao S, Tao S, Rojo de la Vega M, Park SL, Vonderfecht AA, Jacobs SL, Zhang DD, Wondrak GT (2013). The antimalarial amodiaquine causes autophagic-lysosomal and proliferative blockade sensitizing human melanoma cells to starvation- and chemotherapy-induced cell death. Autophagy.

[42] D'Eletto M, Farrace MG, Rossin F, Strappazzon F, Giacomo GD, Cecconi F, Melino G, Sepe S, Moreno S, Fimia GM, Falasca L, Nardacci R, Piacentini M (2012). Type 2 transglutaminase is involved in the autophagy-dependent clearance of ubiquitinated proteins. Cell Death Differ.

[43] Nicotra G, Manfroi F, Follo C, Castino R, Fusco N, Peracchio C, Kerim S, Valente G, Isidoro C (2010). High expression of cathepsin D in non-Hodgkin's lymphomas negatively impacts on clinical outcome. Dis Markers.

[44] Park KS, Kim HK, Lee JH, Choi YB, Park SY, Yang SH, Kim SY, Hong KM (2010). Transglutaminase 2 as a cisplatin resistance marker in non-small cell lung cancer. J Cancer Res Clin Oncol.

[45] Miyoshi N, Ishii H, Mimori K, Tanaka F, Hitora T, Tei M, Sekimoto M, Doki Y, Mori M (2010). TGM2 is a novel marker for prognosis and therapeutic target in colorectal cancer. Ann Surg Oncol.

[46] Deng J, Carlson N, Takeyama K, Dal Cin P, Shipp M, Letai A (2007). BH3 profiling identifies three distinct classes of apoptotic blocks to predict response to ABT-737 and conventional chemotherapeutic agents. Cancer Cell.

[47] Andreasson U, Eden P, Peterson C, Hogerkorp CM, Jerkeman M, Andersen N, Berglund M, Sundstrom C, Rosenquist R, Borrebaeck CA, Ek S (2010). Identification of uniquely expressed transcription factors in highly purified B-cell lymphoma samples. Am J Hematol.

