# Supplementary material for: Dysregulation of autophagy in human follicular lymphoma is independent of overexpression of BCL-2
Source: Oncotarget. 2014 Oct 18;5(22):11653–68. doi: 10.18632/oncotarget.2605 (PMC4294376; doi:10.18632/oncotarget.2605)
Supplement: Supplementary file 1 [file oncotarget-05-11653-s001.pdf]

Dysregulation of autophagy in human follicular lymphoma is independent of overexpression of BCL-2

Supplementary Material

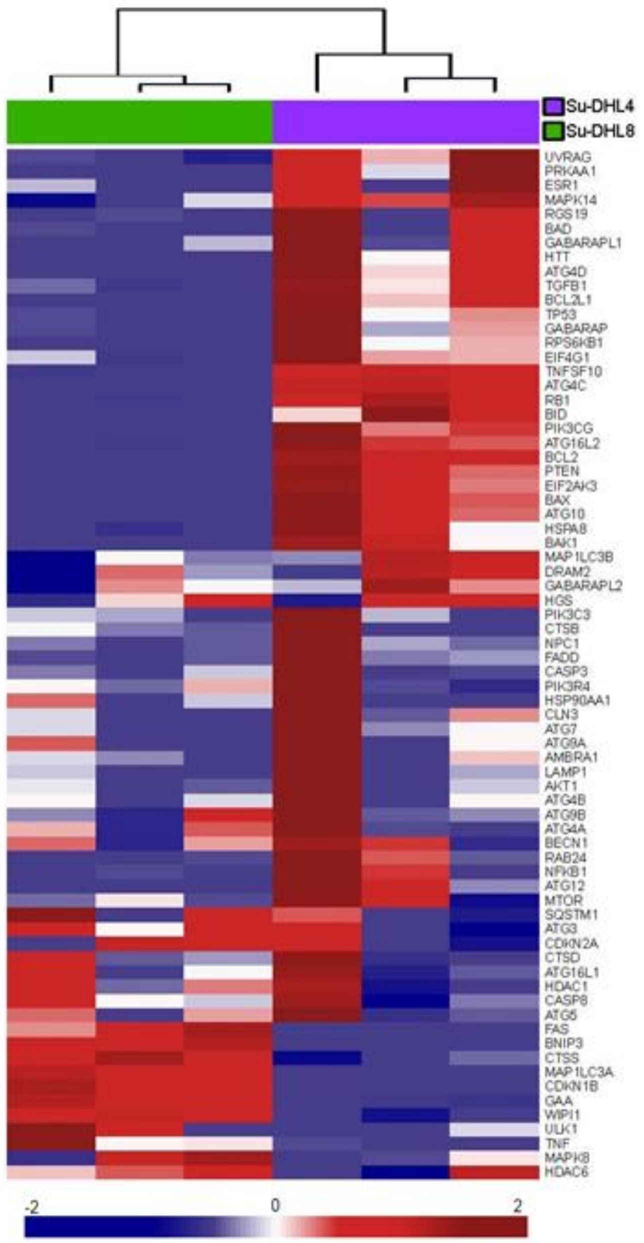

Suppl Figure 1: Unsupervised hierarchical clustering of significantly differentially expressed autophagy machinery and autophagy regulation genes. Heat-map shows triplicate RQ values for Su-DHL4 and Su-DHL8 cell lines.

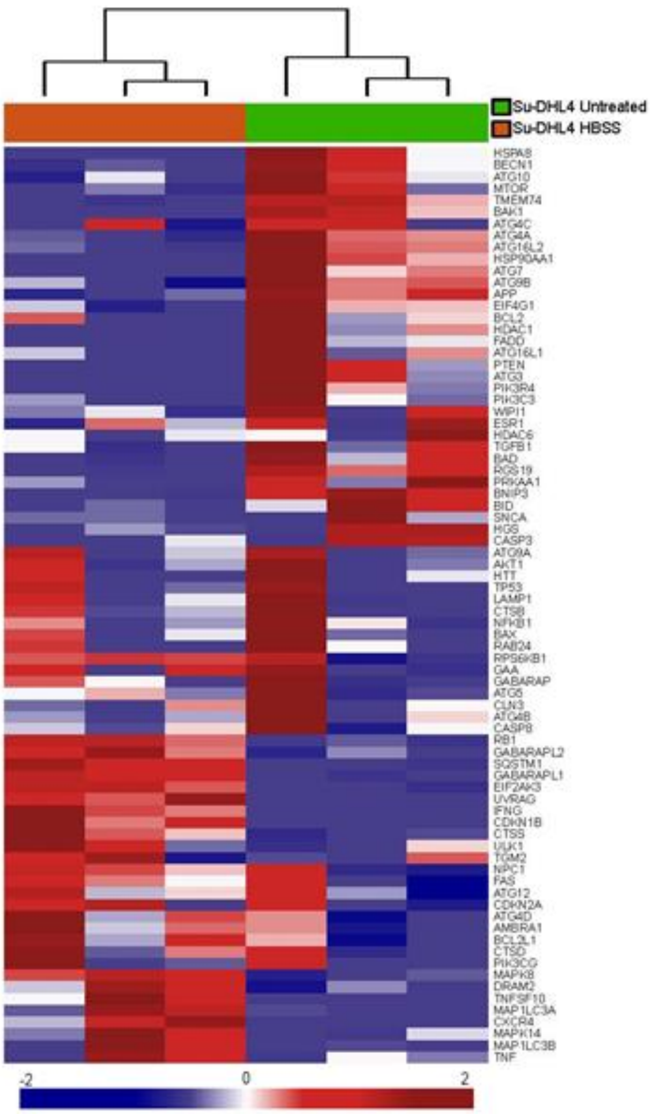

Suppl Figure 2: Unsupervised hierarchical clustering of significantly differentially expressed autophagy related genes in Su-DHL4 cells after incubation in HBSS for 6 hours. Heat-map shows triplicate RQ values for control and HBSS cultured Su-DHL4 cells.

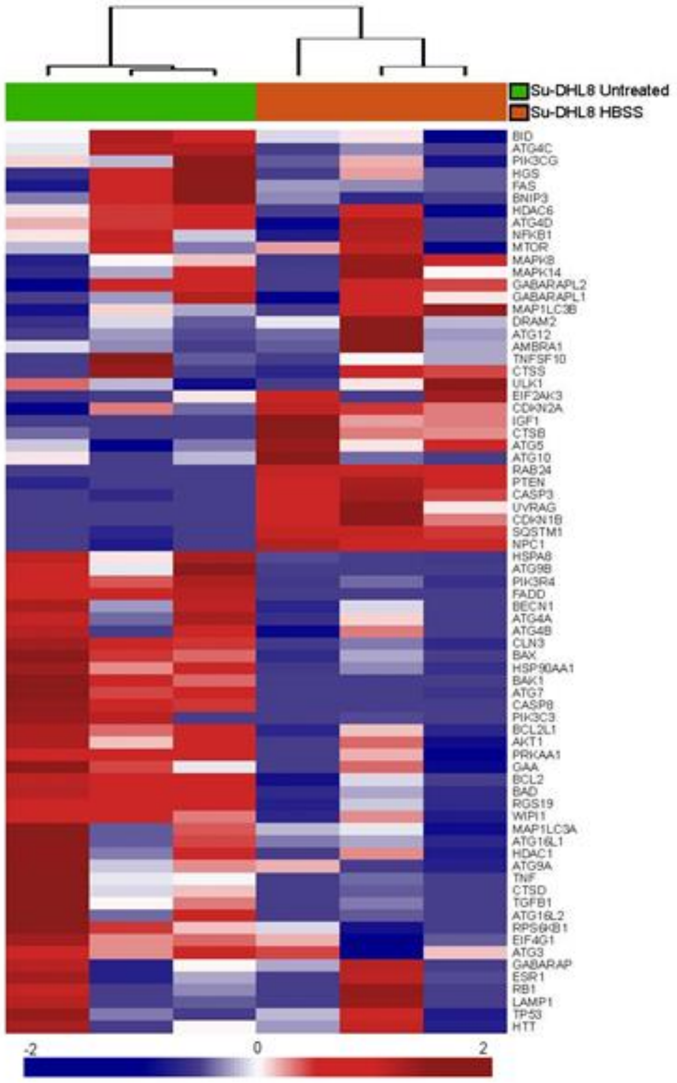

Suppl Figure 3: Unsupervised hierarchical clustering of significantly differentially expressed autophagy related genes in Su-DHL8 cells after incubation in HBSS for 6 hours. Heat-map shows triplicate RQ values for control and HBSS cultured Su-DHL8 cells.

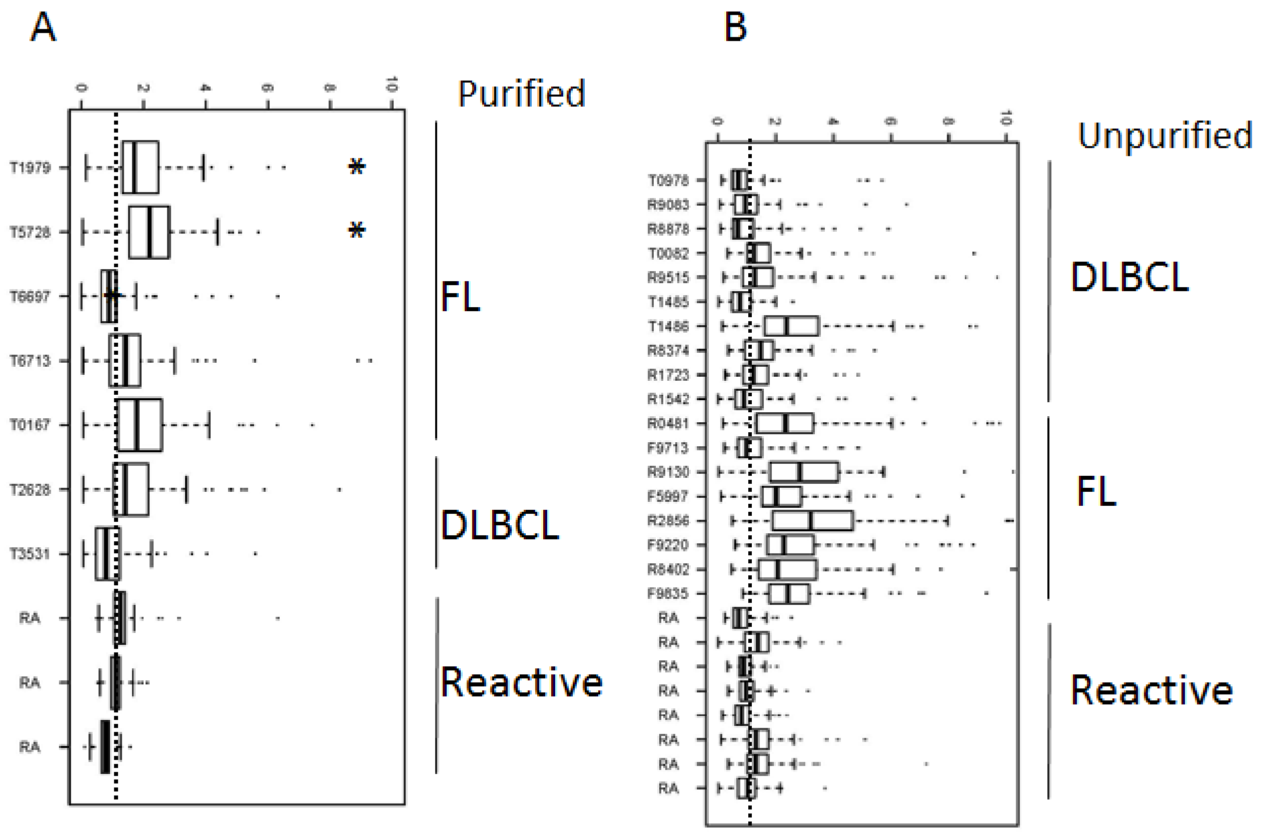

Suppl. Figure 4: Global expression of autophagy genes in purified B cells (A) and unpurified biopsies (B). Data were visualized based on their total global expression levels of those genes included in the un-supervised hierarchical clustering; mid-line represents the median for each sample; box-limits - 25th -75th percentiles; whiskers - 5th - 95th percentiles. RA indicates control reactive LNs. '*' indicates FL samples which transformed in the later stage of the disease. Dashed line reflects median global expression of autophagy-related genes across RA samples.

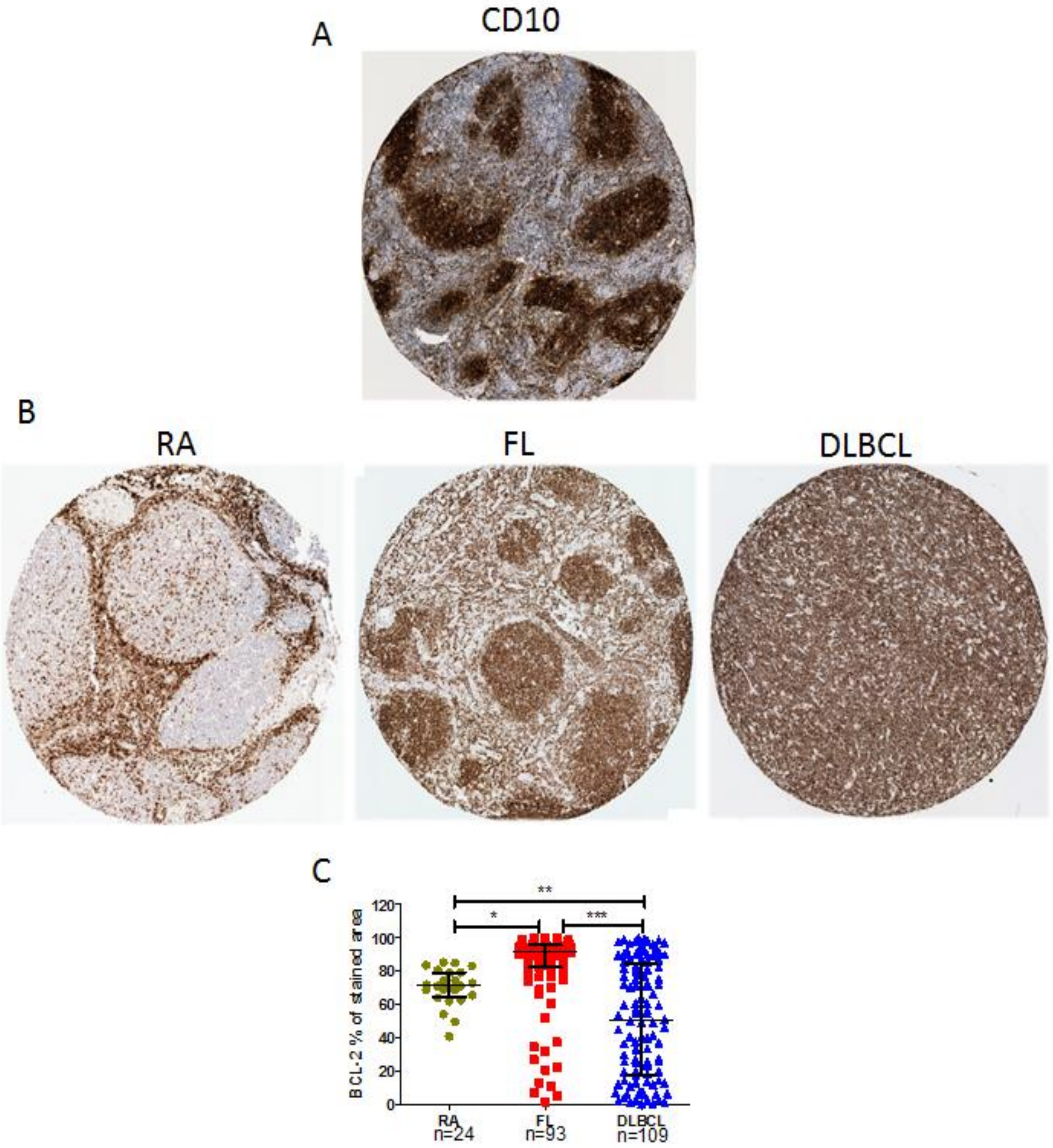

Suppl Figure 5: Histochemical staining of CD10 and BCL-2. (A) CD10 staining of a RA-LN.
(B) BCL-2 staining in RA, FL and DLBCL cores. Brown colors are positive areas for CD10 or BCL-2.
(C) Statistical analysis of BCL-2 expression.

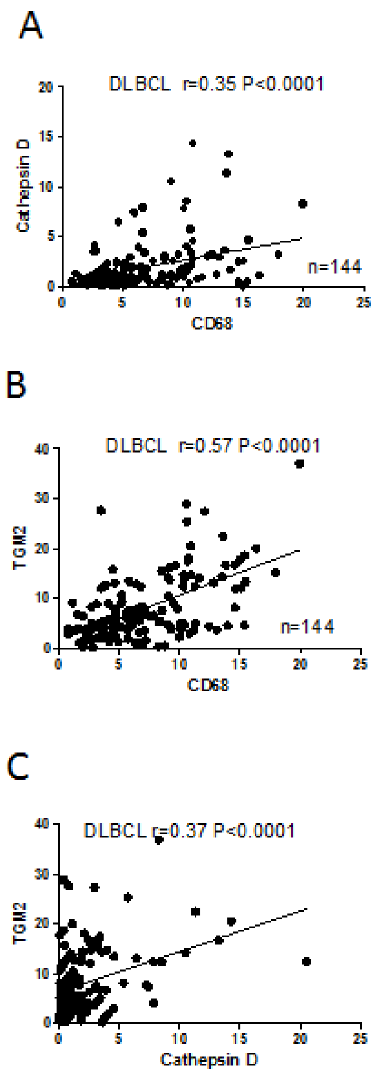

Suppl Figure 6: Correlation between CD68 and Cathepsin D/TGM2. Pearson product-moment correlation method was used to analyze linear correlation between two groups. (A) Correlation Between cathepsin D and CD68; (B) Correlation between TGM2 and CD68; and (C) Correlation between cathepsin D and TGM2. n=144.
