# Supplementary material for: Dysregulation of autophagy in human follicular lymphoma is independent of overexpression of BCL-2
Source: Oncotarget. 2014 Oct 18;5(22):11653–68. doi: 10.18632/oncotarget.2605 (PMC4294376; doi:10.18632/oncotarget.2605)
Supplement: Supplementary file 2 [file oncotarget-05-11653-s002.pdf]

Dysregulation of autophagy in human follicular lymphoma is independent of overexpression of BCL-2

Supplementary Material

Suppl Table 1 : Information on FL patients used for qRT-PCR microarray

Vial ID	Gender	Age at diag	Date of Diagnosis	Date of transformation	Stage[¶]	FLIPI score[§]	Date of the 1st treatment	Response[*]	Date of death	Cause of death[†]
**F9835**	M	56	20/06/1997	15/04/2008	II	1	15/04/2008	Progression	03/12/2008	NHL
**F9220**	M	57	01/04/2001	22/01/2008	IV	3	26/04/2001	CR(U) / GPR		
**R8402**	F	67	22/08/2005	26/04/2011	II	1	14/05/2010	CR		
**T1979**	F	74	01/12/2005	18/05/2009	IV	U	08/05/2009	Progression	08/07/2009	NHL
**T5728**	M	29	15/07/2011	13/02/2012	IV	2	02/03/2012	CR		
**R9130**	F	25	31/05/2007		II	1	04/07/2007	CR(U) / GPR		
**T6697**	M	48	24/03/2011		IV	2	21/03/2012	PR		
**R0481**	F	54	12/04/2002		IV	2	11/01/2005	CR(U) / GPR		
**F5997**	F	63	01/01/1998		IV	3	27/08/1998	Relapse	13/01/2005	NHL
**F9713**	F	89	31/08/2001		IV	2	14/09/2001	N/A	28/09/2002	N/A
**T6713**	M	62	07/03/2012		III	U	N/A			
**R2856**	M	76	06/10/2003		III	2	N/A		20/02/2006	Other
**T0167**	F	74	15/04/2006		IV	U	N/A			

¶ Staging ranges from I to IV with a higher stage indicating more widespread disease. § The Follicular Lymphoma International Prognostic Index (FLIPI) score ranges from 1 to 4 where 1 = low risk (0-1 prognostic factor), 2 = intermediate risk (2 prognostic factors), 3 = high risk (3-5 prognostic factors) and 4 = not assessable; U = unknown. * CR= complete remission; CR(U)= undetermined complete remission; GPR= good partial remission; N/A= not applicable. † NHL= Non-Hodgkin lymphoma; N/A= not applicable.

Suppl Table 2: Information on DLBCL patients used for qRT-PCR microarray

Vial ID	Age at diagnosis	Gender	Date of diagnosis	Stage¶	IPI score§	Date of 1st treatment	Response*	Date of death	Cause of death†
**R1542**	26	F	23/12/2002	VI	1	15/01/2003	CR		
**R8374**	20	M	29/11/2006	V	1	15/12/2006	CR		
**T1486**	33	M	08/01/2009	IV	1	27/01/2009	CR		
**T1485**	55	M	13/01/2009	II	0	20/01/2009	CR		
**T2628**	58	M	23/07/2009	I	0	20/08/2009	CR		
**T3531**	60	M	21/12/2009	III	1	03/02/2010	CR		
**R1723**	55	F	28/01/2003	IV	2	28/02/2003	CR	02/01/2006	NHL
**R8878**	79	F	01/02/2007	IV	4	17/04/2007	Progression	18/03/2008	NHL
**R9083**	33	M	09/05/2007	III	3	15/05/2007	CR(U) / GPR	15/11/2007	NHL
**R9515**	80	M	16/08/2007	IV	4	16/08/2007	Stable disease	19/10/2007	Cardiac failure
**T0082**	18	F	15/01/2008	IV	1	01/02/2008	Progression		
**T0978**	58	M	12/08/2008	IV	4	22/08/2008	Progression		

¶ Staging ranges from I to VI with a higher stage indicating more widespread disease. § The International Prognostic Index (IPI) score ranges from 0 to 5, with 0 indicating absence of all prognostic factors and 5 indicating all prognostic factors are present. * CR= complete remission; CR(U)= undetermined complete remission; GPR= good partial remission. † NHL= Non-Hodgkin lymphoma.

Suppl Table 3: Summary of clinical characteristics of FL patients' samples used for the TMA

	No. of patients	Percentage
	128	100
**Age, years**		
**<60**	77	60.16
**≥60**	51	39.84
**Gender**		
**Male**	64	50.00
**Female**	64	50.00
**Stage at diagnosis**[¶]		
**I**	14	10.94
**II**	14	10.94
**III**	20	15.53
**IV-VI**	71	55.47
**Unknown**	6	4.69
**FLIPI**[§]		
**1 (Low)**	31	24.22
**2 (Intermediate)**	28	21.88
**3 (High)**	34	26.56
**4 (Not assessable)**	17	13.28
**Unknown**	19	14.84
**Proceeded to transformation**	36	28.13
**Time to transformation yr**		
**0-5**	44	34.38[ζ]
**6-10**	37	28.91[ζ]
**>11**	48	37.50[ζ]
**Cause of death**		
**Alive**	51	39.84
**NHL-specific death**	64	50.00
**Unrelated/Other disease**	5	3.91
**Unknown**	8	6.25

¶ Staging ranges from I to IV with a higher stage indicating more widespread disease. § The Follicular Lymphoma International Prognostic Index (FLIPI) score ranges from 1 to 4 where 1 = low risk (0-1 prognostic factor), 2 = intermediate risk (2 prognostic factors), 3 = high risk (3-5 prognostic factors) and 4 = not assessable; U = unknown. ζ Percentages of transformed.

Suppl Table 4: Summary of clinical characteristics of DLBCL patients' samples used for the TMA

	No. of patients	Percentage
	144	100
**Age, years**		
**<60**	84	58.33
**≥60**	60	41.67
**Gender**		
**Male**	89	61.81
**Female**	55	38.19
**Stage at diagnosis¶**		
**I**	13	9.02
**II**	22	15.28
**III**	28	19.44
**IV-VI**	79	54.86
**Unknown**	2	1.38
**B Symptoms¥**		
**Yes**	44	30.56
**No**	69	47.92
**Unknown**	31	21.53
**Serum LDH¤**		
**Normal**	54	37.50
**Elevated**	57	39.58
**Unknown**	33	22.92
**IPI score§**		
**0 (Low)**	23	15.97
**1 (Low)**	37	25.69
**2 (Intermediate)**	28	19.44
**3 (High)**	27	18.75
**4-5 (High)**	9	6.25
**Unknown**	20	13.89
**Cause of death**		
**Alive**	67	46.53
**NHL-specific death**	49	34.03
**Unrelated/Other diseases**	28	19.44

¶ Staging ranges from I to VI with a higher stage indicating more widespread disease; in some cases the stage was not clear at diagnosis and so none was assigned. ¥ B symptoms are weight low, fever and night sweats; yes indicates they were observed in patients; no indicates their absence. ¤ The lactate dehydrogenase value was deemed elevated if it was greater than 480 U/L. § The International Prognostic Index (IPI) score ranges from 0 to 5, with 0 indicating absence of all prognostic factors and 5 indicating all prognostic factors are present; low, intermediate and high refer to risk groups based on IPI scores.

Suppl Table 5: List of primary antibodies and their dilutions

Name of antibody	Type	Company	Cat No	Application	Dilution
**BCL-2**	Mouse	Santa Cruz	sc-509	WB	1:1000
**BCL-2**	Mouse	DAKO	M0887	IH	1:200
**BCL-xL**	Rabbit	Santa Cruz	sc-634	WB	1:1000
**Beclin-1**	Mouse	Santa Cruz	sc-48341	WB<br>IH	1:1000<br>1:250
**GAPDH**	Mouse	Cell Signaling	2118L	WB	1:2000
**LC3B**	Rabbit	Sigma	L7543	WB<br>IH	1:1000<br>1:5000
**SQSTM1/p62**	Mouse	Santa Cruz	sc-28359	WB	1:1000
**SQSTM1/p62**	Rabbit	Abgent	AP2183b	IH	1:7000
**Cathepsin D**	Mouse	Sigma	C0715	IH	1:1000
**CD68**	Mouse	DAKO	M0814	IH	1:8000
**TGM2**	Rabbit	Abcam	Ab421	IH	1:750
**APC-H7-CD20**	Mouse	BD	641396	FC	2.5 μl/10^6 cells
**FITC-CD3**	Mouse	Biolegend	344804	FC	1 μl/10^6 cells
**APC-H7-CD19**	Mouse	Biolegend	302218	FC	2.5 μl/10^6 cells
**PE-CD10**	Mouse	Biolegend	312204	FC	2.5 μl/10^6 cells
**APC-κ light chain**	Mouse	Biolegend	31510	FC	20 μl/10^6 cells
**PerCpCy5.5-λ light chain**	Mouse	Biolegend	316618	FC	5 μl/10^6 cells

FC= flow cytometry; IH= immuno-histochemistry; WB= Western blotting

Suppl Table 6: Genes of autophagy machinery components

Gene code	Full name	Function
**AMBRA1**	Autophagy/Beclin-1 regulator 1	Autophagic vacuole formation
**ATG12**	Autophagy related gene 12	Autophagic vacuole formation/co-regulator for autophagy and apoptosis
**ATG16L1**	Autophagy related gene 16-1	Autophagic vacuole formation/ protein transport
**ATG4 A, B, C, D**	Autophagy related gene 4 A, B, C, D	Autophagic vacuole formation/ protein targeting to vacuole/protein transport/with protease activity
**ATG5**	Autophagy related gene 5	Autophagic vacuole formation
**ATG9A**	Autophagy related gene 9A	Autophagic vacuole formation/protein transport
**ATG9B**	Autophagy related gene 9B	Autophagic vacuole formation
**BECN1**	Beclin-1	Autophagic vacuole formation/co-regulator of autophagy and apoptosis
**GABARAP**	GABA(A) receptor-associated protein	Autophagic vacuole formation/ protein targeting to vacuole/protein transport/linking to lysosome
**GABARAPL1**	GABA(A) receptor-associated protein-like 1	Autophagic vacuole formation
**GABARAPL2**	GABA(A) receptor-associated protein-like 2	Autophagic vacuole formation/protein transport
**IRGM**	Immunity-related GTPase family, M	Autophagic vacuole formation
**MAP1LC3A**	Microtubule-associated protein 1 light chain 3-α (LC3A)	Autophagic vacuole formation
**MAP1LC3B**	Microtubule-associated protein 1 light chain 3-β (LC3B)	Autophagic vacuole formation
**RGS19**	Regulator of G-protein signaling 19	Autophagic vacuole formation
**ULK1**	Serine/threonine-protein kinase ULK1 (ATG1)	Autophagic vacuole formation
**WIPI1**	WD repeat domain, phosphoinositide interacting 1	Autophagic vacuole formation
**ATG10**	Autophagy related protein 10	Protein transport
**ATG16L2**	Autophagy related protein 16-2	Protein transport
**ATG3**	Autophagy related protein 3	Protein transport/ubiquitination
**ATG7**	Autophagy related protein 7	Protein transport/ubiquitination
**RAB24**	RAB24	Protein transport
**DRAM1**	DNA-damage regulated autophagy modulator 1	Linking to lysosome/co-regulator of autophagy and apoptosis
**LAMP1**	Lysosomal-associated membrane protein 1	Linking to lysosome and autophagy induction
**NPC1**	Niemann-Pick disease, type C1	Linking to lysosome
**CTS B, D, S**	Cathepsin B, D, S	Linking to lysosome
**HDAC6**	Histone deacetylase 6	Protein ubiquitination

Suppl Table 7: Genes for autophagy regulation

Gene code	Full name	Function
**AKT1**	v-akt murine thymoma viral oncogene homolog 1	Co-regulator of autophagy and apoptosis
**APP**	Amyloid beta (A4) precursor protein	Co-regulator of autophagy and apoptosis
**ATG5**	Autophagy related protein 5	Co-regulator of autophagy and apoptosis
**BAD**	BCL2-associated agonist of cell death (Bad)	Co-regulator of autophagy and apoptosis
**BAK1**	BCL2 antagonist/killer 1	Co-regulator of autophagy and apoptosis
**BAX**	Bcl-2 associated X protein	Co-regulator of autophagy and apoptosis
**BCL2**	B cell CLL/lymphoma 2	Co-regulator of autophagy and apoptosis
**BCL2L1**	BCL2 like 1	Co-regulator of autophagy and apoptosis
**BID**	BH3 interacting domain death agonist	Co-regulator of autophagy and apoptosis
**BNIP3**	BCL2/adenovirus E1B 19kDa interacting protein 3	Co-regulator of autophagy and apoptosis
**CASP3, 8**	Caspase-3, 8	Co-regulator of autophagy and apoptosis
**CDKN1B**	Cyclin-dependent kinase inhibitor 1B (p27)	Co-regulator of autophagy and apoptosis and cell cycle
**CDKN2A**	Cyclin-dependent kinase inhibitor 2A (p16)	Co-regulator of autophagy and apoptosis and cell cycle
**CLN3**	Battenin	Co-regulator of autophagy and apoptosis
**CXCR4**	Chemokine (C-X-C motif) receptor 4	Co-regulator of autophagy and apoptosis
**DAPK1**	Death-associated protein kinase 1	Co-regulator of autophagy and apoptosis
**EIF2AK3**	Eukaryotic translation initiation factor 2 alpha kinase 3	Co-regulator of autophagy and apoptosis and autophagy induction
**FADD**	Fas (TNFRSF6)-associated via death domain	Co-regulator of autophagy and apoptosis
**FAS**	**Fas** cell surface death receptor	Co-regulator of autophagy and apoptosis
**HDAC1**	Histone deacetylase 1	Co-regulator of autophagy and apoptosis
**HTT**	Huntingtin	Co-regulator of autophagy and apoptosis
**IFNG**	Interferon γ	Co-regulator of autophagy and apoptosis and cell cycle and autophagy induction
**INS**	Insulin	Co-regulator of autophagy and apoptosis
**MAPK8**	Mitogen-activated protein kinase 8	Co-regulator of autophagy and apoptosis
**MTOR**	Mechanistic target of rapamycin	Co-regulator of autophagy and apoptosis
**NFKB1**	Nuclear factor of kappa light polypeptide gene enhancer in B-cells 1	Co-regulator of autophagy and apoptosis
**PIK3CG**	Phosphatidylinositol-4,5-bisphosphate 3-kinase, catalytic subunit γ	Co-regulator of autophagy and apoptosis
**PRKAA1**	Protein Kinase, AMP-Activated, Alpha 1	Co-regulator of autophagy and apoptosis
**PTEN**	Phosphatase and tensin homolog	Co-regulator of autophagy and apoptosis and cell cycle
**SNCA**	Synuclein, alpha	Co-regulator of autophagy and apoptosis
**SQSTM1**	Sequestosome 1 (p62)	Co-regulator of autophagy and apoptosis
**TGFB1**	Transforming growth factor beta 1	Co-regulator of autophagy and apoptosis and cell cycle
**TGM2**	Transglutaminase 2	Co-regulator of autophagy and apoptosis
**TNF**	Tumour necrosis factor	Co-regulator of autophagy and apoptosis
**TNFSF10**	Tumour necrosis factor (ligand) superfamily, member 10	Co-regulator of autophagy and apoptosis
**TP53**	Tumour p53	Co-regulator of autophagy and apoptosis and cell cycle
**RB1**	Retinoblastoma 1	Co-regulator of autophagy and cell cycle
**EIF4G1**	Eukaryotic translation initiation factor 4 gamma, 1	Autophagy in response to other intracellular signals
**ESR1**	Estrogen receptor 1	Autophagy in response to other intracellular signals
**GAA**	Glucosidase, α; acid	Autophagy in response to other intracellular signals

**HGS**	Hepatocyte growth factor-regulated tyrosine kinase substrate	Autophagy in response to other intracellular signals
**MAPK14**	Mitogen-activated protein kinase 14	Autophagy in response to other intracellular signals
**PIK3C3**	Phosphatidylinositol 3-kinase, catalytic subunit type 3	Autophagy in response to other intracellular signals
**PIK3R4**	Phosphatidylinositol 3-kinase, regulatory subunit 4	Autophagy in response to other intracellular signals
**RPS6KB1**	Ribosomal protein S6 kinase, 70kDa, polypeptide 1	Autophagy in response to other intracellular signals
**TMEM74**	Transmembrane protein 74	Autophagy in response to other intracellular signals
**ULK2**	Unc-51 like autophagy activating kinase 2	Autophagy in response to other intracellular signals
**UVRAG**	UV radiation resistance associated gene	Autophagy in response to other intracellular signals
**HSP90AA1**	Heat shock protein 90 alpha class A 1	Chaperone-mediated autophagy
**HSPA8**	Heat shock protein 8	Chaperone-mediated autophagy

Suppl Table 8: Validation of differentially expressed autophagy-related genes.

Gene code	FL				DLBCL			
	F.C. (Array)	P value	F.C. (V)	P value	F.C. (Array)	P value	F.C. (V)	P value
**BECN1**	2.07	<0.005	1.46	0.06			1.23	0.46
**MAP1LC3A**	2.60	<0.005	2.08	<0.05			1.59	0.32
**ATG4B**	4.66	<0.005	2.72	<0.05			1.78	0.13
**DRAM1**	2.38	<0.05	1.05	0.18	4.24	<0.05	5.77	0.05
**CTSD**	2.41	<0.005	2.27	<0.05	4.14	<0.05	8.99	<0.05

Samples used for validation were those previously analyzed in the unpurified PCR array; RA n=5, FL n=4, and DLBCL n=5. Primers used for validation were the same as for PCR array. RPLPO was used as the housekeeping gene. (Array) indicates the initial PCR array and (V) means validation. Validation samples were analyzed in triplicate and the average C_T value of these triplicates used to calculate the RQ value provided standard deviation (SD) was <0.5. In cases where SD was >0.5, data was visualized and if a clear outlier was identified this value was excluded and the average of the remaining duplicates used. Data were normalized to averaged RPLPO, RQ values calculated using the average C_T and the $2^{-\Delta\Delta Ct}$ method as before and FCs calculated as previously described.
